# Stereogenic Centers

**DOI:** 10.3390/molecules23112964

**Published:** 2018-11-13

**Authors:** Alejandro Baeza

**Affiliations:** Organic Chemistry Department and Instituto de Síntesis Orgánica, Universitat d’Alacant, 03690 Alicante, Spain; alex.baeza@ua.es

The demand for chiral organic entities for different industrial purposes has grown exponentially in the last decades. Concerns about controlling the stereochemistry of the new synthesized molecules increased significantly when the European Medicines Agency (EMA) and the US Food and Drug Administration (FDA) launched regulations about the use of chiral molecules not only for pharmaceutical uses, but also for agrochemicals, cosmetics [1].

For these reasons, different methodologies have been developed in order to obtain access to these chiral molecules, with the most straightforward way to achieve them being the creation of a stereogenic center in the molecule. However, this apparently simple task has resulted in being quite challenging over the years. The main strategies developed and employed so far are asymmetric synthesis by using a chiral pool or chiral auxiliaries, the resolution of racemates, and so-called enantioselective catalysis (metal-, organo- and biocatalysis) [2].

In this issue, the majority of the above cited strategies have been gathered together. For example, Kim, Suh and their co-workers reported the total synthesis of fluvirucin A derivative, belonging to a family of antibiotic molecules, which contains 4 sterogenic centers by using the asymmetric synthesis strategy. Thus, starting from an optically active vinylpiperidine as a chiral pool the other centers were established [3]. The kinetic resolution of racemates is also a commonly employed strategy in industrial processes. In this special issue, Nakata, Shiina et al. described the kinetic resolution of racemic 2-hydroxyamides using (*R*)-benzotetramisole as catalyst with high selectivities. The reaction pathway and transition states were studied by means of theoretical calculations [4].

Besides these two examples, the majority of articles disclosed in this special issue are based on enantioselective catalysis. More concretely, there are two articles describing the use of metal-based chiral catalysts for the obtention of stereogenic centers. In this regard, Fernández-Ibáñez, Maciá and co-workers describe the use of a chiral complex between 1,1-binaphthalene-2-*α*-arylmethan-2-ols (Ar-BINMOLs) and Ti(O*^i^*Pr)_4_ acting as a catalyst for the enantioselective addition of organozirconium reagents, generated in situ, to aldehydes. The corresponding alcohols were obtained in moderate to good yields and high enantioselectivities [5]. In the other article related to the use of metal catalysts, Huang and Chang reported the use of an iridium-phosphoramidite chiral complex for the direct asymmetric reductive amination of *O*-protected 3-hydroxyacetophenone as a key step for the total synthesis of (*S*)-Rivastigmine, an active drug against Alzheimer’s disease. The synthesis of the product was obtained in only 4 steps with 82% yield and 96% *ee* [6].

Organocatalysis have emerged as a straightforward and very efficient way to gain access to chiral organic entities; therefore, the exponential grown that this area has experienced in the last two decades is not surprising [7]. As matter of fact, in this issue there are four articles on this topic. Thus, Chinchilla and his group has reported the use of primary amine-salicylamides derived from chiral *trans*-cyclohexane-1,2-diamines as a simple and very efficient organocatalyst for the enantioselective Michael addition of *α*,*α*-disubstituted aldehydes to arylated and heteroarylated nitroalkenes obtaining excellent yields and enantioselectivities for the corresponding adducts. Moreover, theoretical calculations have been also performed in order to elucidate the reaction mechanism [8]. In another work, the group of Huang and Zhang has disclosed the one-pot fluorination and Robinson annulation sequence for the synthesis of densely substituted cyclohexenones using a cinchona alkaloid amine as organocatalyst. The products containing two stereocenters were obtained in good yields, diastereo- and enantioselectivities [9]. Another article dealing with asymmetric organocatalysis has been reported by our group. In this work a *trans*-cyclohexanediamine-benzimidazole derivative acts as bifunctional organocatalyst in the asymmetric electrophilic amination of unprotected 3-substituted oxindoles using di-*tert*-butylazodicarboxylate as the aminating agent. The corresponding products bearing a quaternary stereocenter were obtained in good yields and with good to excellent enantioselectivities [10]. Finally, Herrera and co-workers have disclosed the first organocatalytic asymmetric synthesis of 1-benzamido-1,4-dihydropyridine derivatives through the reaction between preformed hydrazones and malononitriles using *β*-isocupreidine as catalyst. This challenging transformation rendered the corresponding highly functionalized products in good yields and moderate enantioselectivities. [11].

Another strategy has been reported by the Wu and Li group, who combined metal- and organocatalysis for the tandem Ru-catalyzed oxidative dehydrogenation of tetrahydro-*β*-carbolines followed by an aza-Diels–Alder reaction with *α*,*β*-unsaturated ketones facilitated by an aminothiourea organocatalyst. As result, the corresponding indoloquinolizidine-2-ones containing two stereogenic centers, which are motifs present in different naturally occurring alkaloids, were obtained in moderate yields, moderate to good diastereoselectivities, and high enantioselectivities [12].

Another important question when working on the creation of stereogenic centers is the measurement of the optical activity of the new entities. This task is sometimes quite difficult and time-consuming. Therefore, the development of new and reliable tools for the determination of optical purity is highly desirable. In this regard, in this issue Lankhorst’s team reported the use of ^13^C-nuclear magnetic resonance (NMR) for the determination of the enantiomeric excesses of different mixtures of mandelonitrile with the aid of the chiral solvating agent (*S*)-(+)-1-(9-anthryl)-2,2,2-trifluoroethanol (TFAE). As result of this study, it can be concluded that ^13^C-NMR can be a straightforward and efficient methodology for the determination of enantioselectivity in certain molecules [13].

Finally, in this issue a couple of review articles concerning the construction of stereogenic centers are presented. In one of them, Kawanami et al. present recent developments of a well-known procedure, the asymmetric borane reduction of carbonyl compounds using oxazaborolidine catalysts [14]. In the other work, the most recent advances in catalytic (metal-catalyzed and organocatalytic) asymmetric transformations involving the use of oxaziridines, such as oxidation and amination of prochiral molecules as well as cycloaddition and deracemization reactions, in order to generate new stereogenic centers have been comprehensively gathered together by Ren, Yuan and co-workers [15].

In conclusion, it can be said that asymmetric synthesis, in all its aspects, despite being nowadays a well-established field within organic synthesis is still considered a challenging, constantly growing and highly interesting area of research. This assertion is reinforced by the fact that there is an extensive number of articles being published constantly worldwide dedicated to the creation of new methodologies for the construction of new stereogenic centers, or to the improvement of the efficiency or performance of previously reported ones. As guest editor of this special issue, I hope that the selection of articles herein presented can be considered a good reflection of the new findings and the diversity within this exciting topic.
